# A biosocial return to race? A cautionary view for the postgenomic era

**DOI:** 10.1002/ajhb.23742

**Published:** 2022-03-11

**Authors:** Maurizio Meloni, Tessa Moll, Ayuba Issaka, Christopher W. Kuzawa

**Affiliations:** ^1^ Alfred Deakin Institute for Citizenship and Globalisation Deakin University, Geelong Waurn Ponds Campus Waurn Ponds Victoria Australia; ^2^ School of Public Health, Faculty of Health Sciences University of the Witwatersrand Johannesburg South Africa; ^3^ School of Health and Social Development, Faculty of Health Deakin University, Geelong Waurn Ponds Campus Waurn Ponds Victoria Australia; ^4^ Department of Anthropology and Institute for Policy Research Northwestern University Evanston Illinois USA

## Abstract

Recent studies demonstrating epigenetic and developmental sensitivity to early environments, as exemplified by fields like the Developmental Origins of Health and Disease (DOHaD) and environmental epigenetics, are bringing new data and models to bear on debates about race, genetics, and society. Here, we first survey the historical prominence of models of environmental determinism in early formulations of racial thinking to illustrate how notions of direct environmental effects on bodies have been used to naturalize racial hierarchy and inequalities in the past. Next, we conduct a scoping review of postgenomic work in environmental epigenetics and DOHaD that looks at the role of race/ethnicity in human health (2000–2021). Although there is substantial heterogeneity in how race is conceptualized and interpreted across studies, we observe practices that may unwittingly encourage typological thinking, including: using DNA methylation as a novel marker of racial classification; neglect of variation and reversibility within supposedly homogenous racial groups; and a tendency to label and reify whole groups as pathologized or impaired. Even in the very different politico‐economic and epistemic context of contemporary postgenomic science, these trends echo deeply held beliefs in Western thinking which claimed that different environments shape different bodies and then used this logic to argue for essential differences between Europeans and non‐Europeans. We conclude with a series of suggestions on interpreting and reporting findings in these fields that we feel will help researchers harness this work to benefit disadvantaged groups while avoiding the inadvertent dissemination of new and old forms of stigma or prejudice.

## 1 INTRODUCTION: FROM GENETIC TO BIOSOCIAL DETERMINISM? FORWARD TO THE PAST

In race‐stratified societies like the United States, disease susceptibility is often strongly predicted by socially‐defined racial classifications. As one well‐documented example, rates of hypertension are typically 30%–40% higher among African Americans than in other US demographic groups (Benjamin et al., 2019), and there are similar disparities in conditions like diabetes, low birth weight and renal failure (Matoba & Collins Jr, 2017). Among medical and public health practitioners, it is often assumed that these biological differences trace in part to population distributions of genetic variants (Collins et al., 2003). However, extensive research has failed to identify consistent genetic contributors to most race‐related health inequalities, including conditions like hypertension, diabetes, kidney disease and low birth weight (Cerdeña et al., 2021; Cooper et al., 2003; Gravlee, 2009; Kaufman et al., 2015; Williams & Jackson, 2005). Critics of the genetic race concept have traditionally emphasized that a large majority of genetic variation is shared across all continental regions (e.g., Cavalli‐Sforza et al., 1994; Lewontin, 1972; Serre & Pääbo, 2004), while racial group membership is defined based upon cultural, historical, and political criteria specific to each society rather than to ancestry alone (Goldberg, 2016). Social epidemiologists and the environmental justice movement have shown for decades that factors that vary in relation to social‐racial categories, including socioeconomic status (SES), discrimination, neighborhood‐level segregation or the unequal distribution of public benefits or access to care, are strong predictors of disease risk (Bullard, 2008; Williams, 1999), and that statistical adjustment for such factors often attenuates or fully accounts for race‐related health inequalities (Kaufman et al., 1997). These findings have led to a growing consensus among social scientists that race is a social construct that can profoundly shape patterns of health and disease (Goodman, 2014; Gravlee, 2009; Hicks et al., 2014; Krieger, 2011; Leatherman & Goodman, 2020).

In recent years, studies demonstrating epigenetic and developmental sensitivity to early environments, as exemplified by fields like the Developmental Origins of Health and Disease (DOHaD) and environmental epigenetics, are bringing new data and models to bear on these debates (Evans et al., 2021). The DOHaD field explores the environmental sensitivity of prenatal and early postnatal development to long‐term health and disease risk, including via epigenetic changes that influence gene regulation (Gluckman et al., 2010). Because embryonic and fetal development are recognized as critical periods with important long‐term health effects, this has led to a focus on the gestational environment, and maternal experiences like nutrition and stress, as intergenerational determinants of health (Gluckman & Hanson, 2006; Godfrey et al., 2010; Kuzawa, 2005; Kuzawa, 2020). Building on a long history of similar arguments on the social determinants of health (Krieger, 2005, 2011), this emerging science has inspired claims that social exposures, including race‐related inequalities, can drive physiological, developmental and epigenetic processes operating *in utero* and during early postnatal life, becoming “embodied” as relatively durable, albeit in principle modifiable, biological differences (Benn Torres, 2020; Gravlee, 2009; Jasienska, 2009; Kuzawa & Sweet, 2009; Kuzawa & Thayer, 2013).

This challenge to genetic models of race has been welcomed as a potentially important shift towards a fairer society, especially in race‐stratified societies like the United States, where disease susceptibility is often strongly predicted by one's self‐identified or socially‐imposed racial identity. Tracing health differentials to experiences and environments rather than to genes enables opportunities to narrow population‐based gaps in health by shedding light on their underlying social‐structural causes. Moreover, when current health differentials can be understood as partially tracing to past injustices, this also helps connect a group's historically marginalized status to the biological and health inequalities that they experience today (Davis, 2019; Nelson, 2016; Tallbear, 2013).

We agree that epigenetics and other emerging fields in molecular biology enrich our understanding of the social pathways underlying health inequality, and have made contributions to these arguments ourselves (Kuzawa & Gravlee, 2016; Kuzawa & Sweet, 2009). However, as epigenetic analyses of racial/ethnic health disparities expand significantly in scope and impact, we also echo others in urging caution in the interpretation of these new data. Dorothy Roberts, whose 2011 book *Fatal Invention* underscored the ways in which genomic sciences were arbitrating and reifying racial difference, has warned about the racializing potential of epigenetics and the new biosocial sciences: “When scientists write that epigenetic effects of racial discrimination are durable across generations, it sounds perilously close to biological theories of race” (2011: 143; see also Roberts, 2016). Similar concerns of a postgenomic^i^ reinstantiation of race have been raised in a variety of settings and biosocial sciences (Baedke & Delgado, 2019; Duster, 2015; Lock, 2013; Meloni, 2017; Reardon, 2017; Roberts & Rollins, 2020; Saldaña‐Tejeda & Wade, 2019; Saulnier & Dupras, 2017; Tallbear, 2013).

**FIGURE 1:**
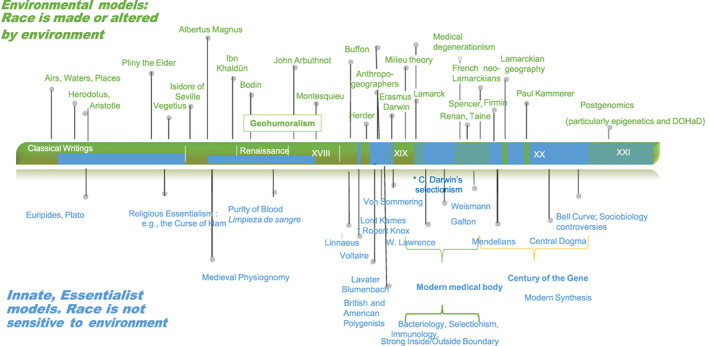
Historical timeline ‐ shifting beliefs in environmental versus fixed models of race in the west. Major scholarly contributions to thoughts about human variation are aligned according to their predominantly environment‐driven (upper/green) and hard/genetic (lower/blue) conceptualization of underlying causes. This historical timeline does not intend to represent the quantitative impact of racialist writings in each historical period (which would be more intense in the period 1750–1940s) but only their historical shifting from an innate to an environmental model over time within the limit of a “Western” perspective. Moreover, as we argue in the article, the separation between environmental and innate models refers to conceptual sources of explanation in racial differences, but this does not preclude that environmental ideas end up supporting assumptions of essential differences between human groups. Finally, while Charles Darwin's selectionism troubles this dichotomy, we have considered for brevity that the overall impact of Darwinian selectionism leans more on the innate model

In this article, we build on this literature to move existing debates ahead in several ways. First, we explicitly focus on contemporary tensions emerging as epigenetic views of race solidify, by exploring historical antecedents in Western beliefs—both scientific and popular—that traced presumed racial differences to environmental, geographic and climatic causes. A deeper excavation of these historical roots underscores that the specific association of racist ideologies with genetic determinism, and the related idea of innateness, was not in the past the most obvious or common way to establish racial differences and hierarchies. Rather, an exclusive emphasis on inborn factors is a relatively recent historical product (See Figure 1). This historical review shows why an emphasis on environmental factors as a driver of group differences is not necessarily benign, and can in fact introduce distortions similar to those more commonly associated with models of genetic race.

Second, we explore how scientific conventions in fields like environmental epigenetics and DOHaD tend to emphasize themes of damage and permanence, along with statistical conventions that encourage binary interpretations of research findings in which effects are described as being either present or not. These practices may, when wedded with assumptions of environmental determinism in racially‐stratified social societies, foster new expressions of typological thinking. As emphasized by earlier generations of geneticists and evolutionary biologists (Lewontin, 1983; Medawar, 1953), a model of phenotypic change based on direct environmental effects—not unlike what we see today in DOHaD and environmental epigenetics—can lead to the assumption that all individuals in a given environment acquire the same structures and adaptations, and depending on understandings of inheritance pathways, possibly even pass these on to succeeding generations. Although these authors were primarily concerned with the impediments that such a process would represent for genetic evolution via natural selection, when applied to humans this line of thought can unwittingly encourage a form of typological thinking in which individuals are reduced to members of a relatively homogenous biological type or essence (Mayr, 1982).

We next conduct a scoping review of scientific work on epigenetics and DOHaD, as applied to issues of race and ethnicity, to assess whether modern variants of environmentally‐driven determinism, typology, normalcy, or essentialism manifest in contemporary research. Our analysis identifies several prominent ways that the concepts of race and ethnicity are treated in this literature, providing us with an opportunity to explore exemplars of each in greater detail. We flag how some of the literature may unwittingly encourage forms of essentialist or typological thinking in which individuals are reduced to their membership in a population deemed of relatively homogenous biology, not because of genes but by virtue of exposure to common experiences as highlighted by specific epigenetic markers. We also highlight the risk that some of this epigenetic literature may implicitly foster normative views about which bodies or environments count as normal or healthy, and against which other groups are implicitly or explicitly viewed as deviating or pathologized (DuBois & Shattuck‐Heidorn, 2021; Wiley & Cullin, 2020). Our effort builds on previous analyses about the potential racialization of epigenetic findings, many of which have interrogated the handling of human diversity and race in narrower treatments of the literature (Mansfield, 2012; Meloni, 2017; Roberts, 2016).

Finally, we conclude with a series of suggestions that we hope will help researchers harness the findings of these new fields to benefit marginalized groups while avoiding the inadvertent dissemination of new forms of stigma or prejudice. The findings from epigenetics and DOHaD have been taken up widely in hopeful articulations within marginalized communities and social justice campaigns (Kowal & Warin, 2018). While in no way intending to dissuade these voices, we echo others in raising concern about the potential for research to contribute to the notion of homogenous and biologically “damaged” communities (Tuck, 2009). In the end, we advocate for a more nuanced accounting of developmental and epigenetic effects and their biological strength, a more explicit consideration of variation and reversibility, and with an emphasis on community engagement across the research process.

## 2 ON THE LONG HISTORY OF BIOLOGICAL DETERMINISM AND RACIALIZATION. THE POWER OF THE ENVIRONMENT BEFORE THE GENE

For many contemporary researchers who grapple with debates about biological race, the modern concept that humans can be arranged into hierarchical typologies is often a starting point for discussion (Biddiss, 1979; Dubow, 1995; Odom, 1967; Stocking, 1987). In the 18th century, the Linnaean system of classifying living things, including humans (Linnaeus, 1735; Sloan, 1995a, 1995b), became the template for later anthropological work that assumed that humans could be ordered into distinct, indelible types that varied in level of sophistication as a matter of inborn potential. Modern racial science, grounded in assumptions of permanent psychophysical differences, experienced new legitimation in simplified understandings of Mendelism and early 20th century anthropology and eugenics. The crux of the argument was that genetic differences, assumed to determine phenotypes in a direct fashion, rendered environmental exposures or habits insignificant when considering racial characteristics (Kuhl, 2002; Weiss, 2010).

This mainstream narrative, which takes post‐Enlightenment scientific developments as its primary reference (see for instance Holt, 2002; Smedley & Smedley, 2018), is not incorrect, but it is certainly partial. Motivated by the understandable concern of linking racism with the modern state powers and settler colonialism (Foucault, 2003; Goldberg, 2016; Graves Jr, 2003; Wolfe, 2016), this narrative has at times isolated modern racial ideology from older ideas of embodied differences among human groups. These older frameworks, even if not “racist” in the modern sense, undoubtedly influenced modern expressions of scientific and popular racism. They helped create an often unnoticeable “mental terrain” (Fields & Fields, 2012) that laid the foundations, and provided an air of cognitive plausibility, for the emergence of modern practices and theories of race.

Inspired by Benjamin Isaac's *The Invention of Racism in Antiquity* (2004; see also 2017), a new wave of historical scholarship (Bethencourt, 2013; Eliav‐Feldon et al., 2009; Heng, 2011, 2018; McCoskey, 2012; Ramey et al., 2015; Whitaker, 2015) has recently challenged the conventional notion that the premodern world, including premodern science (medicine and geography), has little to contribute to understandings of racial thinking (Fredrickson, 2002; Hannaford, 1996; Snowden, 1970). It is not our goal here to discuss the full extent of this ongoing debate in historical scholarship. We are aware of the concern that any expansion of chronological boundaries in considerations of race risks diluting the specific violence of modern racism, the Atlantic slave trade, and European colonialism. We agree that it is better to reserve the word “racism” for modernity, and in our overview, our use of the term race in pre‐modernity may be understood as a synonym of Isaac's term “proto‐racism” (Isaac, 2004; see also Mullings, 2005).^ii^ However, semantics aside, the point we wish to make is substantial. Focusing on a longer history, spanning two millennia rather than three centuries, demonstrates the potential for hierarchy and discrimination to be grounded in, and justified by, patterns of human difference tracing to shared environments and experiences beyond genetic or innate factors.

As a response to the 20th century abuses of genetic determinism, the environment (and nurture) has maintained an allure of progressiveness (Degler, 1991; Pastore, 1949; Toynbee, 1953), especially in the social sciences and humanities (Meloni, 2016). This means that its potential for racialized thought remains hidden. This is particularly obvious in Northern Europe and North America where most of the eugenic movement drew from theories and practices of genetic determinism, and considered an interest in the environment as at best sentimental if not anti‐scientific (from Galton and Pearson in the United Kingdom to Davenport in the United States). However, this lack of interest towards environmental factors is not an inherent property of eugenics or racism but is historically contingent and hence reversible under different circumstances (Bank, 2015; Meloni, 2016; Stepan, 2000). It is thus important to maintain a reflexive stance on these developments, by offering critical tools that help illustrate their potential role as a repository and catalyst for emerging configurations of racial thinking.

### 2.1 Classical antiquity: The power of the physical external environment

Although this was not the only way to construct racial hierarchies in antiquity (e.g., Goldenberg, 2003; Kennedy, 2013: 53–64; Isaac, 2006: 140 and ff.), the tendency to group physical and moral traits of different populations and to associate them to the places where they lived, the air they breathed or the food they ate, was a powerful device to assert the superiority of certain human groups over others in Classical Antiquity (Eliav‐Feldon et al., 2009; Isaac, 2006, 2017).

In an influential passage of the Hippocratic *Airs, Waters and Places* (AWP: second half of the fifth century BCE), Asians are described as “more gentle and affectionate” than Greeks as they live in a land where the weather is uniform and everything grows “more beautifully”. In contrast, in the seasonally‐changing weather of the Mediterranean

the frequent shocks to the mind impart wildness, destroying tameness and gentleness. For this reason, I think, *Europeans are also more courageous than Asiatics*. For uniformity engenders slackness, while variation fosters endurance in both body and soul; rest and slackness are food for cowardice, endurance and exertion for bravery. AWP 23.25‐26: our italics, W. H. S. Jones, Ed).

Albeit overlooked in histories of racializing ideas, *Airs, Waters and Places* is not only a seminal work in medical ecology and the geography of disease, read and translated for centuries in Pagan Antiquity, Latin and Oriental Christendom and the Islamicate, but also one of the first scientific texts to establish “the greatest and most marked differences” (AWP, part 12, Intro) between Europeans and Asians. A whole section of the treatise aims to “compare Asia and Europe, and to show how they differ in every respect, and how the nations (ethneon [*ἐθνέων*]) of the one differ entirely in physique from those of the other” (ibid.).

A generation after Hippocrates, Aristotle built on Hippocrates' ideas with the intent of justifying political differences within a wider imperial framework. People of Asia were now described as “intelligent and skilled but cowardly. Thus they are in a perpetual state of subjection and enslavement.” (350 BCE: Politics, 7.5.6.1327b our emphasis; translation in Kennedy, 2013: 44). It is often claimed that Greek antiquity knew no racism and only indulged in ethnocentric prejudices, mostly based on a dichotomy of Greeks and barbarians (Graves Jr, 2003; Hannaford, 1996). However, even if it would be simplistic to read Aristotle in the light of contemporary concerns (for instance, there are no references to skin color), it is difficult to overlook the racial implications of his dichotomy between Greek political freedom and Asian laziness, passivity, and natural subjugation to tyranny. Filled with references to eugenic topics, the seventh book of the *Politics* (available in the West since 1260) will go on to decisively influence medieval and early modern debates in the Spanish, French and British colonies. There, the Greek/Asian dichotomy will be replaced by one between temperate and tropical weather leading to a climatological distinction between master races and naturally born slaves sealed by the authority of Aristotelian natural philosophy (Huxley, 1980; Hernandez, 2001).

Similar beliefs about the biological embodiment of environments at a population level were far from exceptional or idiosyncratic, and were sometimes supplemented by the idea that customs or social environments could also be inherited. Hippocrates explained the peculiarly lengthened head shape of a certain tribe (called Macrocephali or long‐headed) as the result of “force”, the application of “bandages and other suitable contrivances” at birth when the “head is still tender”. This characteristic however in the course of time had become acquired and “formed naturally, so that usage had nothing to do with it” (AWP, 14: 110–111). Similarly, observing bodies on a military battleground, Herodotus reasoned that Persians' skulls were much softer than Egyptians' because Persians “wear felt hats from birth to shelter themselves from the sun” so that their skulls are “so weak that, if you wanted to strike one with a pebble, you would pierce through it” (cited in Kennedy, 2013: 42).

Ancient racializing patterns crisscrossed boundaries of bodies and environments, nature and culture (Heng, 2018): places were not just embodied but also inherited in ways analogous to the role of “blood” in later times (Wood, 2007). At the same time, the embodiment and transmission of climatic or geographic factors was also viewed as imbued with notions of virtue and nobility or inferiority and servitude (Kennedy, 2013), as one can read in a comment in the pseudo‐Aristotelian *Problemata* (III C BCE) about the connection between extreme climates and brutality of character in African populations (book IV).

The Roman world continued and expanded the climatological tradition. It is common to find typological references to groups based on climatic notions in philosophical writings of the time (for instance Cicero, Seneca, or Posidonius) or in poems. The poet Marcus Annaeus Lucanus (39 CE–65 CE) writes for instance in his poem *Pharsalia*.

*Every* native [*omnis*] of the Northern snows is vehement in war and courts death; but every step you go towards the East and the torrid zone, the people grow softer as the sky grows kinder (emollit gentes clementia caeli) (Lucan, 8.363–6, our italics)

For the Romans, this way of envisioning the homogeneity of environmentally‐shaped group characteristics was more than a matter of casual speculation: They expanded it to practical areas such as architecture and military science. Numerous classifications of the military skills of different populations were made as a result of the direct impact of physical or social environments on bodies and minds (Geltner, 2019; Meloni, 2021). Based upon these principles, the blood and bravery of different ethnic groups, as shaped by their climatic or social conditions, were used to distinguish the best troops to recruit for specific military tasks (whether mere force or ingenuity were required; Irby, 2016). In the most‐read military treatise of Roman late antiquity and the Middle Ages (*De re militari* or *Epitome of Military Science*), Vegetius (late IV century CE) evokes environmental stereotypes as important criteria when recruiting troops. People from near the sun, being parched by great heat, are more intelligent but have less blood, and therefore lack steadiness and confidence to fight at close quarters. On the other hand the peoples of the north, remote from the sun's heat, are less intelligent, but (…) readiest for wars. Recruits should therefore be raised from the more temperate climes (Book I, Section II “From What Regions Recruits Should be Levied”).

With Roman historians like Tacitus or Livy we also see the appearance of a certain asymmetry in how negative and positive environmental effects are perceived as impacting populations, which foreshadows later doctrines of racial purity: men transplanted from Rome into inferior locales “acquire the degenerate characteristics of the alien environment” but the reverse is only rarely mentioned (Kennedy, 2013: 33 and ff.; Isaac, 2006). Similarly, even when not directly related to climatic factors but historical ones, slavery is often portrayed as having long term enfeebling and corrupting effects that are no longer reversible (Tacitus, Agricola 11. 4–5 (2014); late 1st Century CE). Several of these stereotypes—including a marked bipolarity between one's own “median” environment as optimal, and with extreme places/climates as deviating from normality—will persist or reappear with modern colonialism and slavery and go on to influence Enlightenment ideas of race and aesthetic norms (Bindman, 2002).

### 2.2 Medieval and early modern conceptions: Innate but changeable

The Middle Ages are generally an overlooked period for racial and ethnic classification based on the incorporation of environmental effects, but their influence on later medical and geographical thinking that influenced the mental cartography of early modern European colonialism and political theory was immense (Bartlett, 2001; Weeda, 2016). In particular, after the eleventh century, with the translation of Greek, Persian, and Arabic medical treatises in the Latin west, notions of direct environmental effects on group characteristics started to substantively shape medieval writings. Historians and geographers built on this set of assumption to produce one of the first full‐fledged forms of ethno‐typology, which made claims about essential group differences that traced to differences in birthplace or latitude (Weeda, 2017). Not only were people seen as a mirror of where they lived, but human groups who differed by “blood” were often thought to inherit the same traits if living “under the same sky” (Weeda, 2017: 98).

From the 12th century onwards, changes in religious and political formations led to an increasing tendency in the middle ages to essentialize biological differences in humoral complexion following emerging ideas of human nature, heredity or religious affiliation (Biller, 2001; Boureau, 2008; Resnick, 2012). However, medical and geographical treatises of the time still allowed for factors that could potentially alter the innate but changeable complexion of human groups. These included migration (sometimes described as transplanting to another soil), wet‐nursing and, albeit more difficult to achieve in the space of one generation, religious conversion (Resnick, 2012: 12). The first two factors in particular would go on to become key components of early modern anxieties about racial regeneration and degeneration. The medieval notion that “everything generated in a place, derives its natural properties from that place” (Albertus Magnus, cited by Bartlett, 2001) deeply shaped anxieties surrounding the first colonial expansions and lasted well into the European Renaissance and Elizabethan England (Floyd‐Wilson, 2003). Even if we take modern colonialism as a starting point for a full deployment or hardening of racial thought it is impossible to disconnect the geographical, medical, and anthropological assumptions of early modern colonialists from the antecedent premodern science that shaped and informed their practices and theories. Columbus' turn to the Tropics for instance largely relied on geographical and cosmographical assumptions about the (moral/geographic) notion of latitude elaborated during the late Middle Ages (via philosopher Albertus Magnus, 1200–1280; and, later, geographer Pierre D'Ailly: 1351–1420). Such notions established a geographical hierarchy across the globe between the colonizers, who were deemed capable of self‐governance due to their temperate location, and the colonized, who were viewed as incapable of self‐rule owing to the perceived simplicity of life in the warm tropics (Wey‐Gómez, 2008).

Besides the impact of religious discourses, early modern views of race were also influenced by medieval understandings of human biology and disease that traced individual and group traits to geography and place. Hippocratic‐Galenic ideas of environmental effects on humors led to concerns about the “transplantation” of human groups into new soils and under new stars that “would affect not only themselves but their descendants” (Feerick, 2010). Hence, colonies became places where the colonizer could be “re‐raced” (ibid.: 19) spurring anxieties around the potential degeneration of a nobler European “stock” under new environmental conditions (food, waters, stars: Earle, 2012; Baedke & Delgado, 2019). Medical views also shaped emerging racial classifications in Spanish America, with the humoral temperament of each racial group (Native, Creole, Spanish) defined by a complex combination of geographic, climatic and nutritional factors (Canizares‐Esguerra, 2006; López Beltrán, 2007). As in later periods (Meloni, 2016, chapter 4), this malleability of race formation was both contrasted and reinforced by colonial rules that helped solidify hierarchical relationships between different groups (Earle, 2003, 2010).

In early colonial contexts, racial science began to intermingle fixed and malleable characteristics in a strategic way, particularly in debates on acclimatization. The capacity for response and change among European races was contrasted with the perceived imperviousness to novel experiences in other groups, along a precise hierarchical line that foreshadows 20th century ideologies of genetic racial purity (Bethencourt, 2013; Osborne, 2000). The initial expectation that descendants of the first black slaves would progressively lighten their skin in northerly countries after several generations gave way to the notion that, while White could degenerate into Black in hot climates, the reverse process was impossible (Delbourgo, 2012; Fredrickson, 2002; Jordan, 2013). In general, the narrative of acclimatization tended to oscillate between an optimistic hope for the adaptability of people to new areas and the reinforcement of a moralistic connection of people and places between latitude and lassitude (Bale, 2002; Livingstone, 1991).

### 2.3 Ideas of environmental effects within modern racial science

Some historians have argued that it was precisely this fear of changeability under new environmental conditions, and the tendency of these ideas to discourage colonial projects, that incentivized their replacement by notions of race as innate and immune to such environmental effects (Braude, 2011). Whatever the case, it is important to remember that the post‐Enlightenment emergence of fixed racial typologies did not displace premodern environmental models of race, but in many cases updated and reused them within emerging evolutionary frameworks.

The relationship between modern racial science in the 18th and 19th centuries and earlier expressions of environmentally‐driven human difference, as reviewed above, can be divided into three phases or historical developments. First, at the very foundation of the Enlightenment in the 18th century, we find a strong presence of environmental and climatological explanations of race differences. Old Hippocratic and Aristotelian arguments were reframed by Enlightenment intellectuals, particularly in areas like medical topography, becoming the “linchpin to understanding most eighteenth‐century pronouncements about the body's appearance” (Golinski, 2010; Livingstone, 2012). It would therefore be somewhat artificial to view the history of racism as the brainchild of the Enlightenment and overlooking the reality that Enlightenment intellectuals themselves wittingly inherited their ideas from Greek and Roman sources. Montesquieu (d. 1755) is an obvious case in point here. Several of the arguments we find in his *Spirit of Laws* (1752) tracing human differences to inherited environmental effects are hardly new in the light of our longue durée history. For instance, the connection between hot climates and despotism and cold climates and liberty or “intrepid actions”; or the notion that certain groups (Tartars for instance) would carry with them their “servile spirit” which they had “acquired in the climate of slavery” (1752/1914: 353, 355). Although he was opposed to slavery as contrary to reason, Montesquieu nonetheless found it if not justified at least comprehensible within certain climatic contexts:

“there are countries where the excess of heat enervates the body, and renders men so slothful and dispirited that nothing but the fear of chastisement can oblige them to perform any laborious duty: slavery is there more recognizable to reason” (Chapter VII, *Another Origin of the Right of Slavery)*.

This and similar arguments that “the effeminacy of the people in hot climates, has almost always rendered them slaves” (VII, 2) did not go unnoticed and were selectively adjusted to the purpose of justifying slavery in French and American colonies (see for instance Hughes, 1750; Ghachem, 1999; Livingstone, 2012).

A second development, and one well‐described in histories of racism (Gould, 1996), relates to the rise of polygenism, or the idea that human races have distinct origins (which by itself in terms of origin myths goes back to antiquity and flourished already in medieval times), and the telescoping of racial differences into anatomical observations of group differences that were viewed as innate and impervious to environmental factors. The tables of skull measurements in the writings of Samuel George Morton (d. 1851) left little room to environmental causes, which were now understood as an alternative to inborn biology—unlike premodern understandings of human diversity that tended to mix nature and culture rather than bifurcate them (Heng, 2018). While not entirely denying the role of climate, late eighteenth century and early nineteenth century polygenists had little patience for environmental explanations of diversity espoused by the monogenist school. A century later, physical anthropologists would similarly dismiss as “sentimental” the notion of environmentally‐driven, plastic influences on biophysical characteristics as advanced by Boas (1912) in his study of immigrants (1912, and foreshadowed, albeit in relative obscurity in Anglophone anthropology, by Haitian philosopher and anthropologist Anténor Firmin, 1885, 2002). Since then, it is fair to say in countries like the US, England and Germany, that classical hereditary determinism has trumped alternative explanatory frameworks based on external factors (Bowler, 1994; Haller Jr, 1970; Stocking Jr., 1968). Undoubtedly, and to simplify, the mainstream story of nineteenth and early twentieth century racism and eugenics understood the proponents of biological race as holding to hard, genetic hereditary views, such as those espoused in books like *The Bell Curve* (Herrnstein & Murray, 2010).

A third historical development involved the revitalization of environmental explanations of racial differences via Lamarckian evolution (Bowler, 1984; Meloni, 2016). Particularly salient in France's Third Republic (1870–1940), in the Soviet Union, in Fascist Italy, and in Latin America (see respectively: Schneider, 1990; Adams, 1989; Cassata, 2011; Stepan, 2000), but also in British and Australian eugenics (Bowler, 1984: Wyndham, 2003), different strands of pessimistic Lamarckism emphasized the notion that the “accumulated burden of past negative environmental influences had created a thoroughly degenerate heredity that was difficult to improve rapidly” (Adams, 1989, p. 121). At a theoretical level, the persistence of environmental modes of racial differences well into modern evolutionary debates finds its roots in the work of Herbert Spencer (1820–1903), who was Lamarckian in evolutionary views, and Social‐Darwinist in policy orientation. A deep believer in the inheritance of acquired characters, Spencer adhered to a hierarchical vision based upon what he interpreted as population differences in plasticity and the capacity to learn and evolve as a society. In his 1876 *Comparative Psychology of Man*, Spencer described the “relative plasticity” of different human races, with what he viewed as the most developed (i.e., European) being the “most plastic” with the others being “characterized by a greater rigidity of custom than (…) the more civilized nations of the West” (Spencer, 1876: 304 and ff.). This double standard—progress for some but not for everyone—often gave rise to a mixture of optimism and pessimism in neo‐Lamarckian discussions of race and social progress. When it came to non‐White groups the “behaviors carved into the savage's system by Lamarckian inheritance” could not be easily overpowered by education and social learning” (Jackson & Weidman, 2004: 83). A connection of Lamarckism with recapitulation theory by authors such as Haeckel or the American Neo‐Lamarckians was similarly used to claim that colonial populations were “immature or underdeveloped forms of humanity” (Bowler, 1994: 159).

Similar Lamarckian thinking, melded with rising social stratification and inequality, fueled concerns about decay and degeneration in the European metropoles (Pick, 1993). Referring explicitly or loosely to Lamarckian ideas, doctors, biologists, educators, psychiatrists, anthropologists and social reformers highlighted the direct pathogenic impacts of urban squalor, moral vice, or insalubrious climate on the mental and moral characteristics of whole social groups, their germplasm and progeny (Meloni, 2016). The rediscovery of Mendel's work in 1900 did not eclipse transitional notions of race in which ancestral conditions of life directly shape heredity, which persisted well into the 1920s and 1930s (Meloni, 2016; Paul, 1994). Early twentieth century work in American paleontology and geography for instance illustrates the persisting influence of Lamarckian ideas, and with them of older climatological explanations, which were harnessed to make sense of the characteristics of local cultures and ways of living as a response to the “impression” of their “surrounding conditions” that are then “transmitted to their progeny” in the words of American paleontologist Nathaniel Shaler (1841–1906) (Campbell & Livingstone, 1983: 271; Peet, 1985 similar trends for French geography: Archer, 1993; Loison, 2011).

### 2.4 Historical lessons for current work in postgenomics: DOHaD and epigenetics

Our historical review underscores how intrinsic differences between groups were historically traced to the durable effects of environments and shared experiences, with the exclusive emphasis on fixed biological characteristics a comparably recent phenomenon. The lessons from our historical evidence—that there are many templates for biological racism, or, more accurately, racisms in the plural (Bethencourt, 2013)—concurs with and reinforces the social science argument that racism is a “moving target” and a “scavenger ideology” (M'Charek, 2013; Solomos, 1996). Without flattening different historical contexts, legal infrastructures and political economies into a simplified continuity, we suggest that it is possible to highlight a number of recurring characteristics in environmentally‐patterned models of human difference. First, a predominance of typological models based on the causal power of the environment—where common biological essences are viewed as being directly established by environmental effects, and ignoring within‐group variability. Second, binary thinking manifests in several ways. Environments were divided into categories of normal (that of the observer) and abnormal/pathological (that of the colonial subject or “other”), and “exposures” were similarly viewed as having effects that were either present or absent, ignoring the possibility of a spectrum of phenotypic outcomes. Thirdly, there was a tendency to establish an asymmetry between negative and positive environmental effects, with the former more common and used to capture the developmental trajectory of non‐Western or subordinated groups. The overly pessimistic skew and focus on marginalized communities was epitomized by the use of the word “degeneration”; plasticity or environmental influences were appreciated mostly in the negative. Fourthly, this work often assumed that environmental and social disturbances were transferred directly to individual bodies which are portrayed as passive recipients of external forces: damaged environments (or non‐European ones) were viewed as becoming *ipso facto* damaged bodies thus eliding a wider focus on underlying causes. Finally, it was common to argue that environmental factors can cause loops that are difficult to break, with whole groups being stuck in social or cultural inertia because of acquired environmental insults.

Of course, even when based on environmental models, contemporary expressions of environmentally‐ or socially‐patterned race and biology do not extrapolate seamlessly from these recurring patterns and historical examples. Any convergence between current models and the pre‐modern ideas that we discuss is unfolding despite the unique confluence of political, economic, and scientific realities that inspire contemporary postgenomic scholarship around race. Most obviously, work in fields like DOHaD and environmental epigenetics has the explicit goal of clarifying the causes of preventable disease. Overwhelmingly, it is good intentions—to clarify pathways, reduce societal impacts, and address the unequal distribution of ill‐health—that motivate this work. However, as notions of genetic race are in some quarters challenged by epigenetic and developmentally‐grounded frameworks, some of the conventions of biomedical research may create openings to unwittingly recapitulate typological and essentialized thinking (Mansfield & Guthman, 2015; Roberts, 2011). Or to put our argument in different terms: if the challenge to genetic determinism advanced by postgenomic research in areas such as epigenetics or microbiomics reveals newly‐appreciated biological pathways that are sensitive to environments and experiences, can we anticipate the re‐inscription of pre‐modern ideas of environmentally‐driven human difference at a molecular level? To answer this question, we set out to investigate the literature and findings in DOHaD and environmental epigenetics that address the role of race/ethnicity in human health.

## 3 A SCOPING REVIEW OF ENVIRONMENTAL EPIGENETICS, DOHAD, AND RACE/ETHNICITY

### 3.1 Introductory observations

Before considering the specific role of DOHaD and the related field of epigenetics in debates about race and health, it is worth considering some of the practices that are common in these fields in general. Most human studies of the long‐term development or epigenetic effects of early experience have used observational designs in which there is extensive potential for confounding because key exposures and influences on health, such as environmental stressors, diet, or activity levels, tend to cluster as a result of influences like socioeconomic status, ethnicity, class, or gender (Hernan, 2018). Some of the most highly cited and discussed attempts to work around these limitations have harnessed natural or quasi‐experimental designs, such as by evaluating the impacts of maternal exposure during pregnancy to “exogenous” stressors like a war‐imposed famine, terrorist attack, global pandemic, or earthquake (LaPlante et al., 2008; Roseboom et al., 2006; Torche, 2011). Because this work approximates a randomized exposure, it achieves a stronger basis for causal inference, but it does so at the expense of studying severe shocks and stressors. This focuses attention on the effects of unusual or dramatic exposures that are not generally targets for policy or intervention. They are also less capable of addressing any beneficial effects of enrichment or favorable exposures that may be less amenable to study as an exogenous shock. These issues also apply to experimental animal model studies in the DOHaD field, which often impose relatively extreme prenatal nutritional stress on species with far less maternal capacity for fetal nutritional buffering than humans (Kuzawa & Thayer, 2013; Thayer et al., 2020).

In addition to using models of severe and dramatic stress, relatively little work to date has been explicitly designed to clarify the potential reversibility of early life effects (Reiss et al., 2019). This creates a default assumption that any effects induced by these (again, often severe) exposures are also permanent. While of course not every study can address reversibility, the overwhelming skew towards demonstrating hardened effects while not exploring the possibilities of long‐term plasticity, amelioration, or reversibility creates the impression of permanent scarring. This simplified picture may be further reinforced by the common convention in biomedical research of reporting relationships in a binary fashion, as being present or absent, depending on whether a threshold for statistical significance has been reached (Wasserstein & Lazar, 2016). While there has been a strong push to do away with a focus on binary or “bright line” assessments of the significance of findings in fields like biostatistics and epidemiology (Cummins & Marks, 2020), in fields like DOHaD and environmental epigenetics, this practice remains common and risks reinforcing the idea that whole populations faced with early life adversity and stress experience negative “programming” and carry “biological baggage” or “scarring” as a result of those experiences (Escher, 2018; McEniry, 2013; Roseboom et al., 2021), a notion that can take on wider and more complex significance when applied to the issue of race‐based health inequity (Singh et al., 2019; McEwen et al., 2019: 7). As Dorothy Roberts again has warned, the new “biosocial” sciences offer the potential to document the harms of unequal structural violence; however, when devoid of context and relying on simplified causal models, they also risk obscuring wider political relationships that cause harm and even perpetuate the notion of inequality “as a product of flaws in peoples' bodies” (2016: 127).

These concerns are real, but are they warranted? How common are essentialized and typological notions of environment‐driven race and human difference in the DOHaD and environmental epigenetics literatures? While an important catalyst for studies of developmental plasticity, DOHaD remains a niche in a wider trend exploring relationships between epigenetic changes, particularly DNA methylation (DNAm), and racial/ethnic differences. Within this broader field, do we see an emphasis on environmental determinism, a focus on negative environments understood as leading to permanent scarring, or perspectives that foster binary interpretations of exposures and outcomes? And does a focus on newer and more technologically‐driven biological understandings of the pathways of environmental sensitivity divert attention away from socio‐structural causes of health inequality (Roberts, 2016)? To answer these questions, we have conducted a scoping review following consolidated methodologies in the field (Arksey & O'Malley, 2005) and using the PRISMA‐ScR (Preferred Reporting Items for Systematic reviews and Meta‐Analyses extension for Scoping Reviews) checklist (Tricco et al., 2018). The two guiding research questions in our review are: (a) are DOHaD or epigenetics related studies addressing racial/ethnic differences in human health?; (b) and if so, do these studies implicitly reflect or reiterate some aspects of the environmentally‐driven template (determinism, typology, normalcy, or essentialism) that we explored in the historical section? A scoping review was deemed appropriate to address our questions (Grant & Booth, 2009). It allowed us to map emerging themes in a large literature and provide a narrative analysis of their conceptual contributions without compromising the reliability of study findings due to contrasting methods across the studies (Arksey & O'Malley, 2005; Tricco et al., 2018).

### 3.2 Study design, search strategy, and eligibility criteria

**FIGURE 2:**
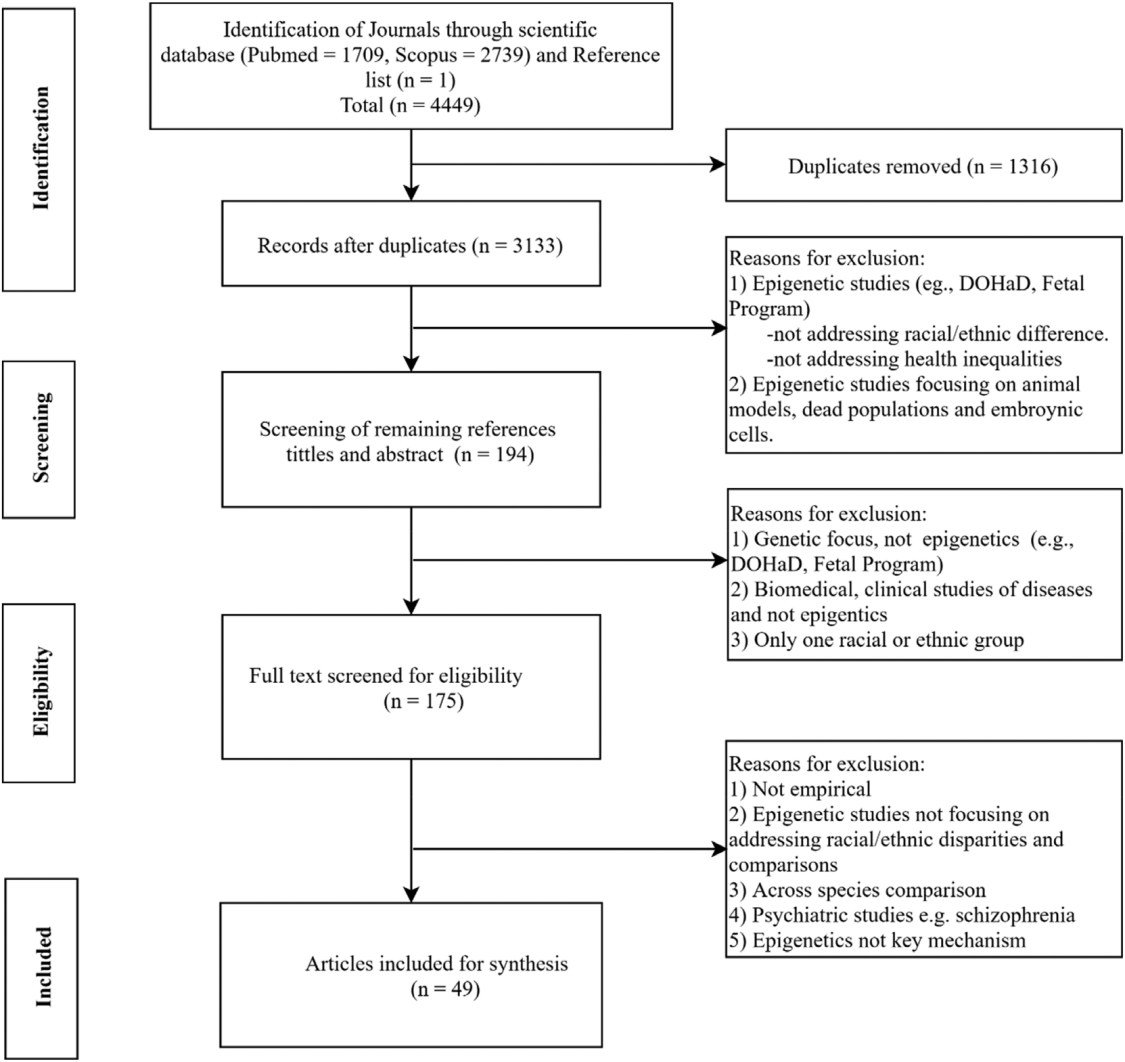
PRISMA flow diagram for the study selection process

Our systematic search of the English literature was carried out using two electronic databases, PubMed and Scopus, from inception to October 20, 2021. MeSH terms were combined by Boolean commands “AND” and “OR” in the search: “epigen*” OR “DOHaD” OR “Fetal program*” OR “Barker Hypothesis” OR “Developmental origin*” OR “DNA methylation*” OR methylat* OR “Developmental program*” OR “Developmental plasticity” AND “race*” OR “raci*” OR “ethnic*” OR “continental population group*” OR “ancest*”. We manually searched reference lists of the included studies and similar reviews for additional articles. Studies were considered for eligibility if they met the following criteria (a) peer‐reviewed empirical studies reporting quantitative data; (b) human rather than animal studies relating to environmental factors and racial/ethnic differences; (c) focusing on at least one of the epigenetics or postgenomic elements (e.g., DOHaD, DNA methylation, Fetal program, Barker hypothesis, developmental plasticity); and (d) including a comparison between two or more racial/ethnic groups. Studies were excluded if they (a) focused solely on genetic factors rather than epigenetics; (b) addressed strictly medical areas and specific diseases with no relevance for discussions about race and racial differences in health; or (c) were psychiatric studies, which constitute a largely distinct literature, or reviews, commentaries, letters, and gray literature. In conclusion, a total of 49 epigenetics‐related studies addressing racial/ethnic differences in human health met all inclusion criteria.^iii^ The PRISMA flow chart outlines the selections process, and exclusion and eligibility criteria (Figure 2). Table S1 summarizes the characteristics of each study in our final review sample.

### 3.3 Synthesis of main findings

**FIGURE 3:**
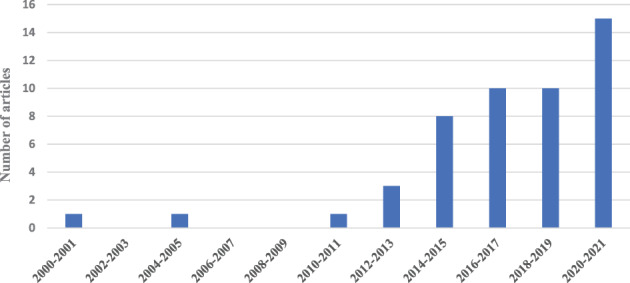
Shows the increase over time of epigenetics and epigenetic related studies (DOHaD, barker hypothesis, fetal origins, developmental origin, developmental plasticity) addressing racial/ethnic differences and health inequalities in the postgenomic era that we have selected following our inclusion and exclusion criteria

The growth over time of publications addressing epigenetics/DOHaD and race/ethnicity is evident from our search. While between 2000 and 2011 published studies increased only by 2%, increase was 8% between 2012 and 2017, 21% between 2018 and 2019, and finally 29% since 2020 (see Figure 3).

Of the study designs, longitudinal and cohort studies made up a large portion (67%) while 27% were cross‐sectional studies and 6% were population‐based case–control studies. In terms of geography, the United States accounted for the lion's share of authors (38 studies, 77%), with an emerging presence, however, from other countries after 2018: United Kingdom (6%), South Africa (4%), China (4%), Poland (2%), India (2%), Mexico (2%), and Qatar (2%) (see Table S1, column 1 and Table S2). The studied populations largely mirrored the geographic affiliations of the authors: a large proportion of the 49 studies focused on health inequality within racially defined US African American (66%) and Hispanic American (22%) samples. Other studies addressed Indian (2%), Chinese (2%), and South African ethnic divisions (4%), while a study from the UK addressed admixed individuals from Colombia (Rawlik et al., 2017), and one from Poland (Daca‐Roszak et al., 2020) focused on differences between samples of European and Chinese ancestry.^iv^ In terms of sex and age, more than half of the participants were of mixed sex (both men and women) (53%), followed by studies on women only (27%), men‐only (10%), and infants and children (10%).

Despite heterogeneities in methodology and geographic focus, all 49 studies mirror the growing perception that epigenetic markers, and particularly DNA methylation (98%; along with one including RNA non‐coding, Li et al., 2020), are a potential basis for explaining ethnic or race‐based differences in health, disease incidence, aging, or reactions to exposures or drugs (Adkins et al., 2011; Barfield et al., 2014; Davis Lynn et al., 2019; Heyn et al., 2013; Li et al., 2020; Needham et al., 2015; Rai et al., 2019; Rawlik et al., 2017; Song et al., 2015). As one study claims, “DNA methylation diversity is a source of variability in human groups at macro and microgeographical scales” (Giuliani et al., 2016; similarly McKennan et al., 2020) and, given that it is considered “highly divergent between populations” (Fraser et al., 2012) can be used to elucidate variation in biological traits or different effects of environmental exposures on racially defined populations: That is, using the terminology of these studies, African American, European, Caucasians (sic), Hispanic, Chinese, or Western (see Table S1, column 3 for complete overview). Even when inter‐population differences in DNAm are considered small by authors, in one study they are described as representing “a possible source of markers that could be used for human population stratification” (Daca‐Roszak et al., 2020: 2). Epigenetic factors are considered a “possible mechanism” driving observed phenotypic differences not just between individuals but also “among different ethnic groups” (Rawlik et al., 2017) but this does not necessarily imply a recognition of direct environmental causation or epigenetic inheritance only. From this angle, it is possible to divide our selected studies into three categories. A first, larger group (58%) remains agnostic about causation of epigenetic differences or refer to, for instance, a “multifactorial combination” or “complex interplay” between genetic and environmental exposures (for instance, Adkins et al., 2011; Chitrala et al., 2020; Davis Lynn et al., 2019; Fraser et al., 2012; Lapato et al., 2018). A second group instead minimally mentions the environment, which is often framed as “lifestyle”, and with a predominant tracing of DNAm variability to underlying genetic factors. These articles, accounting for 16% of the selected sample, tend to be critical of the widely presumed responsiveness of methylation to environmental factors, with one typical study concluding that “race/ethnicity‐dependent blood DNAm levels in particular, and blood DNAm levels in general, are primarily driven by genetic factors” (McKennan et al., 2020: 663; see also Barfield et al., 2014; Chitrala et al., 2020; Conway et al., 2015; Heyn et al., 2013; Mozhui et al., 2015; Philibert et al., 2020). Finally, 13 articles (26%) assume the possibility of direct environmental effects (e.g., socio‐economic factors, food, lifestyle, diet, and toxins) on the epigenome (Demerath et al., 2015; Do et al., 2021; Galanter et al., 2017; Horvath et al., 2016a; Jhun et al., 2021; Lynn et al., 2019; McKennan et al., 2020; Park et al., 2018; Rawlik et al., 2017; Tajuddin et al., 2019; Wang et al., 2016). It is a common assumption among these groups, however, that more research is needed to establish the relative contributions of genetic, environmental or epigenetic factors (Adkins et al., 2011; Barfield et al., 2019; Davis Lynn et al., 2019; Enokida et al., 2005; Jhun et al., 2021; Needham et al., 2015; Straughen et al., 2015; Tehranifar et al., 2018).

Given the largely medical nature of the reviewed literature, an emphasis on pathology is predominant, and exposures are generally understood exclusively in the negative, that is, as a source of risk for chronic disease and mortality and dysfunction of biological processes (Agha et al., 2016; Chan et al., 2017; de Mendoza et al., 2018; Do et al., 2021; Kader et al., 2020; Jhun et al., 2021; Lara et al., 2020; Lapato et al., 2018; Needham et al., 2015; Pheiffher et al., 2020; Rizzo et al., 2020; Tekola‐Ayele et al., 2020; Tehranifar et al., 2018; Wang et al., 2018; Tsegaselassie et al., 2021; Zhu et al., 2016).

With one exception (Horvath et al., 2016b), among studies that explore environmental influences on the epigenome, the imprint understood as being left by the environment include deleterious and harmful factors like pathogens, cigarette exposure, psychosocial stress, smoking, an adverse *in utero* environment, and poverty (de Mendoza et al., 2018; Demerath et al., 2015; Heyn et al., 2013; Mozhui et al., 2015; Wiley et al., 2013; Zaghlool et al., 2018). Populations emerging from biomedical categories are now reframed as manifesting through differences in methylation level: “African American adults”, “African American children”, “black women”, “black ethnicity”, “Hispanic ethnicity”, “Native Hawaiians”, “Kazak diabetics”. All these groups are conveyed as having abnormal methylation levels and are hence defined as at‐risk populations, even in instances when the data do not obviously fit with this account (e.g., higher global methylation levels, suggesting reduced cancer risk, in African American children; Chan et al., 2017; Mozhui et al., 2015; Okosun et al., 2000; Paredes‐Céspedes et al., 2021; Straughen et al., 2015; Tehranifar et al., 2018). An emphasis on interpreting outcomes in binary terms, with effects discussed as significant or not and without further consideration of effect sizes or uncertainties is also seen throughout the studies considered in this sample. All 49 (100%) of the studies used *p*‐values of 0.05 in their analysis as a threshold to arbitrate whether a significant difference exists between groups (e.g., Agha et al., 2016; Kader et al., 2020; Salihu et al., 2016). In some instances, the authors evaluate the strength of the effect or consider issues like statistical power and measurement reliability as a constraint on statistical significance, but in general this practice is rare (Chan et al., 2017; Giri et al., 2017, discussed below in more details).

Only three articles (6%) mention or recognize the importance of wider socio‐structural factors as “drivers of racial health differences” (Pepin et al., 2021; see also Lynn et al., 2019, Tsegaselassie et al., 2021). Reversibility is explicitly highlighted by 14 articles (28%) but most discussions of this are brief and often limited to the conclusion, with occasional reference to prospective pharmaceutical or therapeutic interventions (Chan et al., 2017; Demerath et al., 2015; Devaney et al., 2015; Enokida et al., 2005; Lynn et al., 2019; Okosun et al., 2000; Pepin et al., 2021; Pheiffer et al., 2020; Rai et al., 2019; Salihu et al., 2016; Tajuddin et al., 2019; Wang et al., 2016; Wiley et al., 2013; Zhu et al., 2016).^v^ One significant exception is a study by Giuliani et al. (2016) that emphasizes reversibility and variability as a theme throughout the article.

If we look at the longer history of racial differences via environmental effects examined in this article, two studies stand out for an explicit recognition that shared exposures are direct drivers of biological differences defined in terms of race/ethnicity. The first, from the United States (Galanter et al., 2017), investigates the extent to which differences in DNA methylation between Latino sub‐groups (Mexican and Puerto‐Rican) could be explained by their shared genetic ancestry vs. experience. The study claims that even “after adjusting for ancestry, significant differences in methylation remained between the groups at multiple loci, reflecting social and environmental influences upon methylation” (2017: 3). Although this language unproblematically assumes that variance in outcomes predicted by markers of genetic ancestry index the effects of these genes, rather than of correlated social and environmental exposures, this study does argue that some of the variance in methylation associated with ethnicity “may be due to environmental exposures correlating with global ancestry” (2017: 15). A second study, from India (Giri et al., 2017), is explicit about the importance of having one basal methylome map for each population and the potential value of epigenetic marks as distinct criteria for racial classification beyond and sometimes in contrast to genetic findings. Looking at DNAm profiles from the two larger ethno‐linguistic groupings in India (Indo‐Europeans and Dravidians) the study compares them with (previously published) data from other “global populations”: Japanese, Caucasians and African Americans. Two results emerge, as the study claims, “in partial contrast” to known genetic differences. First, that Indians (Indo‐Europeans and Dravidians) have “a distinct methylation profile” compared to other global populations and despite their recognized differences in allele and genotypic frequency: for the authors, this is interpreted as evidence for epigenetic sensitivity to environmental effects: “This indicates that people living in more or less the same climatic zone, with similar life style pattern(s) and a similar socio‐economic background may have similar methylation at the global level” (2017: 658). Second, they describe epigenetic similarities between the Indian and Japanese samples. For the authors: “This is in contrast to earlier genetic studies” that showed “a distant relation” between Japanese and Indian populations. Their conclusion is again that “similarities between Indian and Japanese methylome” may be a consequence of similar “lifestyle and food habit and also similarities in cultural beliefs in India and Japan”. They also note that both populations “are predominantly starch eating” (2017:660).

This and other examples highlight that, while often associated with a promise to disrupt fixed or typological identities (Malabou, 2016), an in‐depth look at the way in which racial categories are operationalized in epigenetics research points to a more complex scenario. Only a limited number of studies are self‐reflective about uncritical usage of racial categories, observing that “the use of race as a clinical proxy could perpetuate implicit racism in the healthcare setting” (Pepin et al., 2021: 2075). Although most studies build on pre‐existing ethnic classifications for biomedical research (as highlighted in Table 1), they show little introspection or challenge to the validity and processes of social construction of these categories (one exception is Philibert et al., 2020). While several studies find racial classification biologically meaningful (Barfield et al., 2019; Chitrala et al., 2020; Conway et al., 2015; Daca‐Roszak et al., 2020; Lara et al., 2020; Park et al., 2018; Tajuddin et al., 2019), a few in particular stand out for noting that “methylation differs significantly by race” (Davis Lynn et al., 2019; Enokida et al., 2005), and one (Heyn et al., 2013) claims to be able to perfectly separate distinct populations (Caucasian‐American, African‐American, and Han Chinese‐American) on the basis of differences in methylation. The language of population, and intra‐group variability in biological responses to environmental exposures, is rare (one exception is Giuliani et al., 2016). Sources of heterogeneity—such as immigration status, levels of income or the wide array of meanings, countries, and backgrounds coalescing under different racial categories(Hispanics for the United States, or South African “Black” and “Colored” for instance) are rarely acknowledged (for instance, Demerath et al., 2015). A tendency to use typological language occurs even when race is recognized to be a poor proxy for human physiology (Jhun et al., 2021; Mozhui et al., 2015; Pepin et al., 2021). Visual representations of racial categories within several articles do not help convey inter‐individual variability, with racial average traits being produced in a way that glosses over within‐group heterogeneity (Barfield et al., 2019; Heyn et al., 2013; Pheiffer et al., 2020).

A final example in our sample involves the use of epigenetic clocks, which use measures of genome‐wide methylation to gauge the pace of biological aging (Ryan, 2021), to racial/ethnic differences in outcomes like all‐cause mortality and cardio metabolic disease (Horvath, 2013). In a highly cited article in our sample the authors remain cautious about the mechanisms by which “race/ethnicity and sex affect molecular markers of aging” (Horvath et al., 2016b: 171). At the same time, the study adopts several conventions that reify typological thinking around human population variation. Like much scholarship in this literature, the article relies on broad racial divisions (Caucasians, Hispanics, African Americans) and describes differences across these groups in largely typological terms, without devoting space to intra‐population heterogeneities (e.g., “African Americans have been shown to have longer telomere lengths than Caucasians”; “Hispanics have a consistently lower IEAA compared to Caucasians”; “Tsimane have a lower intrinsic aging rate than Caucasians” (2016: 170). Because the epigenetic clock employed by this study was developed using methylation data derived from studies conducted primarily in European‐descent populations, epigenetic age acceleration in all groups is gauged against this “universal” clock which is hence set as the norm from which others deviate (2016). The typological framings of this article were amplified in the popular media, with Hispanics portrayed not as an at‐risk population but as the carriers of some anti‐aging secret, a potential “fountain of youth” at the molecular level (Johnson, 2016).

## 4 FOSTERING A BALANCED APPROACH IN POSTGENOMIC TREATMENTS OF RACE

Scholarly critique of DOHaD and epigenetics has thus far largely focused on the structural imbalances in gender discourses (Richardson & Stevens, 2015; Sharp et al., 2018), which can lead to phenomena like mother blaming and a shifting of the focus of root causes from social injustice to individual agency (Pentecost, 2018; Richardson et al., 2014). Echoing others (Baedke & Delgado, 2019; Keaney, 2021; Lock, 2013; Meloni, 2017; Roberts 2011 and 2016; Roberts & Rollins, 2020; Saldaña‐Tejeda & Wade, 2019; Saulnier & Dupras, 2017) we feel that it is also crucial to encourage introspection on the intersections of these fields with conceptions of biological race to mitigate any unintended consequences that these newer lines of investigation may have. In our critical review, we certainly do not intend to convey that epigenetic or DOHaD approaches emphasizing environmental influences are only negative in their impact, and indeed, as we have emphasized, these fields are helping stimulate crucial new understandings of the social and historical pathways underlying health inequalities. Moreover, even research interpreted through a lens of scarring or damage can be geared, depending on the context, towards fostering community resilience (Müller & Kenney, 2021). Indigenous, black and marginalized communities have similarly leveraged biosocial understandings of historical trauma as part of an agenda of social justice to argue for reparations (Hatala et al., 2016; Kowal & Warin, 2018; Warin et al., 2020).

Our call for caution should in no way be read as dismissing the potential value of new understandings of biological complexity in attempts to redress ongoing racial disparities in health and well‐being. At the same time, we must remain vigilant about minimizing an enduring legacy of what Indigenous academic Eve Tuck refers to as “damage‐centered research”, which documents pain and injury without corresponding inquiry into issues like resilience or reversibility (Tuck, 2009). As Tuck emphasizes, research often catalogues harms with the well‐meaning intention of producing change but in practice rarely catalyzes changes to the material or political causes of those harms. Instead, this focus may leave populations with the label and self‐perception of damage, thus perpetuating a modern variant of inborn hierarchy even if framed with compassionate intent.

We feel it is important to maintain vigilance around the assumed role of these pathways and how we interpret their effects, and echo recent calls that care is needed in fields like epigenetics to ensure that this work does not end up causing unintended harm to the communities that it seeks to benefit (Benezra, 2020; Delgado & Baedke, 2021; Roberts & Rollins, 2020). Indeed, the general trends that we identified in our review of this literature—including the reification of typologies via flattened and homogenized racial groups, the lack of attention to variation and reversibility, and the adoption of DNA methylation as a potential marker of racial classification—open up opportunities to label groups as pathologized or impaired owing to environmental effects that are perceived as not only durable and semi‐permanent, but generalizable to entire groups. Our exploration of the history of pre‐modern racial typologies shows that environmentally‐determined notions of human types have been used in flexible ways to justify and buoy systems of structural inequality and oppression for centuries. In the spirit of moving beyond critique, we end with recommendations for ways that researchers currently working in these fields can help ensure that their work benefits communities while avoiding any unintended stigma or repeating the simplifications and pitfalls of the past.

### 4.1 Positive steps

Our discussion above points to practices that could help minimize the unintended stigmatizing of groups, including: (a) moving away from interpretations of data that reinforce simplified cause‐effect models, (b) avoiding characterization of outcomes as present or absent, (c) and avoiding the generalization of pathologies to entire groups without considering the magnitude, heterogeneity, or reversibility of these effects. In addition to these common features of studies in the DOHaD and environmental epigenetics fields, which run the risk of painting a simplified picture of permanent and multi‐generational scarring, these practices are often further reinforced, as we discuss above, by the common scientific convention of reporting relationships in a binary way, as being “present” or “absent”, depending on whether a threshold for statistical significance has been reached by a given study. In a recent article, Wasserstein et al. (2019) aspire to a world “beyond '*p* < .05'”: “This is a world where researchers are free to treat “*p* = .051” and “*p* = .049” as not being categorically different, where authors no longer find themselves constrained to selectively publish their results based on a single magic number” (2019:1). Many of these methodological concerns echo those raised in Non's (2021) recent review of social epigenomics: publication bias may favor studies that show wide methylation differences, but that may not translate to important phenotypic difference; the difficulties in isolating DNA methylation from other variables (genes, timing, environments); the challenges in measuring social exposures (such as discrimination); sampling biases favoring white populations (which perhaps feeds into the use of white populations as the norm‐see below); and limited collaboration with social scientists. The predominant focus in DOHaD research on documenting exposure‐disease relationships that are characterized in such a de facto binary fashion can reinforce the idea that populations faced with early life adversity and stress necessarily carry negative biological baggage as a result of those experiences. These arguments apply to the fields of DOHaD and epigenetics generally and are not limited to their application to issues of health disparities. However, we feel that they have particular salience when applied to address race‐based health inequity, because they have the potential to slot back into historic norms of viewing race as an essential, immutable category that individuals are born with, and that characterizes the health and societal potential of entire populations.

### 4.2 Beyond normality and embracing plasticity, variation, and reversibility

The concerns that we raise here mirror recent calls for critical self‐appraisal across the social sciences more generally. As one important example, a recent special issue in this journal (Cullin et al., 2021) scrutinized traditional normative assumptions in human biology. In the Pearl Memorial Lecture that inaugurated the special issue, Wiley (2021) outlines how cultural assumptions of normality intersect with and reinforce statistical norms, and vice versa. Described as “ethno‐biocentrism” (Wiley, 2021), Wiley points to a number of research habits that reinforce the slippage between statistical and cultural normality (including reinforcing whiteness as the norm): the adoption of “normal ranges” of biomarkers derived from research in high income populations of predominantly European ancestry; and how a trait's frequency influences views about its acceptability and value. The well‐cited article within our review that explored population‐level differences in epigenetic aging (Horvath et al., 2016b) used a multi‐tissue epigenetic clock derived from samples of primarily European descent and groups of varied ancestries were then compared to one another against this normative center. This type of practice runs the risk of ascribing the notion of abnormality to marginalized communities (Graves Jr, 2021; Mansfield, 2012; Mansfield & Guthman, 2015). With many outcomes portrayed as biologically fixed and the majority of studies exploring negative causes, we worry that DOHaD and epigenetic science may inadvertently contribute to the reification of racial typologies as essential and internally homogenous.

In this context, besides intra‐population variability, future work should place greater emphasis on exploring not just the development of resilience from early adversity (Vassoler et al., 2013) but also the reversibility or amelioration of early life effects in response to later favorable experiences or other interventions. When reversibility is not explored, the default of permanence may often be assumed, thus increasing the potential for stigmatization. In fact, ethnographic work on vulnerable communities exposed to epigenetics and DOHaD knowledge has highlighted growing “bottom‐up” demand for research into practices that build resilience, rather than pursuing additional work on damage and adversity (Müller & Kenney, 2021). There are encouraging trends already underway in epigenetics research (Gapp et al., 2016), and some of these findings have also influenced the DOHaD‐related literature (Taouk & Schulkin, 2016). As increasingly recognized in all corners of the social sciences (for anthropology: Lock, 2015; Thayer & Non, 2015), avoiding neomolecular reductionism goes hand in hand with a meaningful engagement with the communities that are subjects of study. Adopting the adage “Nothing about us, without us” will provide an important safeguard to help ensure that this work is conducted in ways that are in service of the needs and interests of participant communities (Bader, 2019; Lock et al., 2022; Wallerstein et al., 2017).

In light of the entanglements between scientific findings and historical, social and political forces, collaborative and interdisciplinary endeavors will continue to prove essential to any future efforts to improve the production, interpretation and consumption of epigenetic knowledge (Müller et al., 2017). We are reminded that post‐WWII genetics was able to disentangle itself from some of its darkest racist applications only as a result of intense exchanges and collaborations with anthropologists, sociologists, and historians which lead to a more humanistic, and realistic, view of race (Smocovitis, 2012). If we can apply the metaphors from this field to its own development, early exposure to cross‐disciplinary collaboration should help foster critical introspection and a stronger mature science. We hope that this article, and the meeting of disciplines represented by us as authors, will help to nurture this project.

## CONFLICT OF INTEREST

The authors declare no conflicts of interest.

## Supporting information


**Table S1** Characteristics of Included Studies.

**Table S2** Supplementary Information.

## Data Availability

The data that support the findings of this study are openly available in PubMed at https://pubmed.ncbi.nlm.nih.gov/.

## Endnotes

^i^
Postgenomics is an increasingly common umbrella term that covers all research on the complex molecular architecture that connects genomic sequences to the phenotype, inclusive of a new set of approaches dubbed the “‐omics” (e.g. epigenomics, microbiomics, transcriptomics: Richardson & Stevens, 2015). While it goes beyond the goals of this article to discuss the multiple semantics of the concept (Griffiths & Stotz, 2013; Keller, 2015; Landecker & Panofsky, 2013; Meloni, 2016), we abstract one specific aspect of this multifarious term, that is the emerging logic of *imprint* by which the experiential and physical milieu “originating outside the body” is seen as impacting directly the biological body producing “shifts in conformation deep inside cells” (Lappé & Landecker, 2015: 153). Environmental epigenetics and DOHaD are particularly representative of this biosocial exchange between inner body and outer environment. It is at this level that we see our longer history of racial thinking driven by environmental factors as speaking to present concerns.

^ii^
Positing an early beginning for protoracism in Western philosophical, geographical and medical thinking has nothing to do with the evolutionary psychology claim that racism is somehow an in‐built, species‐wide cognitive mechanism (see for a critique, Jackson, 2017). We believe instead that a better way to criticize this naturalizing approach is to look at the longer history of practices and imperial/colonial racist thought, which at least in the West has its origin with Greco‐Roman imperialism (Isaac, 2017).

^iii^
The screening process has followed these steps: Authors 1 and 3 screened the title and abstract as well as full text for study eligibility. In the process, disagreements were discussed and resolved by consensus between author 1 and 3, and when there was further disagreement authors 2 and 4 were involved for final decision. Data extraction and charting were conducted by authors 1, 2 and 3. Extracted data include the study reference, study aims, country where the study was conducted, study design, definition of racial/ethnic groups, causal role of environmental factors in shaping ethnic differences, and whether or not studies discuss reversibility‐or amenability to intervention.

^iv^
It is important to note that multiple studies used data from a single data source, particularly from the USA, and may explain in part the higher number of studies conducted from that country. For example, three US studies used data from the Gene Expression Omnibus (GEO) database (Daca‐Roszak et al., 2020; Galanter et al., 2017; Giuliani et al., 2016), while two studies used data from the “Conditions Affecting Neurocognitive Development and Learning in Early Childhood study” (CANDLE) (Mozhui et al., 2015; Philibert et al., (2020), and another two from the Healthy Aging in Neighborhoods of Diversity across the Life Span data (HANDLS cohort study) (Chitrala et al., 2020; Tajuddin et al., 2019). Two studies conducted in the UK utilized the Twins UK cohort study data (Do, W. L., et al., 2021; Enokida, H., et al., (2005), while a study each from the US (Lara et al., 2020) and China (Li, J., et al., 2020) also utilized the Surveillance, Epidemiology, and End Results (SEER) program data. For further clarification, percentages of the participants and study characteristics are included in Supplemental Table S2.

^v^
Theranifar et al., (2018) is difficult to categorize in the reversibility group: the topic is briefly discussed but long term persistence of effects of exposure is also highlighted. Zaghlool et al. (2018) does not discuss reversibility although it references experimental approaches highlighting reversibility.
