# Supplementary material for: A biosocial return to race? A cautionary view for the postgenomic era
Source: Am J Hum Biol. 2022 Mar 11;34(7):e23742. doi: 10.1002/ajhb.23742 (PMC9286859; doi:10.1002/ajhb.23742)
Supplement: Supplementary file 1 — Table S1 Characteristics of Included Studies. [file AJHB-34-0-s001.docx]

| **Author/study location** | **Study aims** | **Participants (Racial/ethnic groups as defined in the study)** | **Study design** | **DNA methylation (DNAm) as racial/ethnic signature** | **Discussion of environmental causation** | **Reversibility-Amenability to Intervention** |
| --- | --- | --- | --- | --- | --- | --- |
| **Adkins et al., (2011), USA** | Identifying if differences in DNA methylation in adulthood are also present at birth | 201 African American (N = 107) and Caucasian newborns (N = 94) | Longitudinal cohort | Various racial groups differ in terms of their patterns of DNA methylation | Only minimal and speculative | Not Discussed |
| **Agha et al., (2016), USA** | Identifying underlying molecular markers (particularly DNA methylation) associated with fetal growth | Multi-ethnic cohort of pregnant women and their children. (White = 338), (Black = 56), (Hispanic = 37), (Other = 45). | Prospective study | Neutral | Adverse in-utero environment can influence epigenetic patterns and affect fetal development: reference to programming; | Not Discussed |
| **Barfield et al. (2014), USA** | Illustrating the potential for confounding due to population stratification by demonstrating widespread associations between DNA methylation and race | 388 individuals African American (N= 365) and Caucasian (N = 23) | Cohort study | Widespread associations between DNA methylation and race | Differences could arise from epigenetic inheritance or population specific environmental factors, but most likely genetics | Not Discussed |
| **Barfield et al. (2019), USA** | Exploring the association between DNAm and daytime sleepiness | 619 individuals: African Americans (AAs), 202 Hispanic Americans (HAs), and 285 European Americans (EAs), | Longitudinal Cohort | Differences in methylation may explain some of the differences in sleepiness between AAs and EAs | Prospective: need for additional research that links key aspects of an individual’s environmental and sociological exposures with epigenetic markers and health conditions | Not Discussed |
| **Chan et al. (2017), USA** | Examining the association between DNA methylation socioeconomic status, asthma severity and race/ethnicity | Americans (5–21 years, N = 41.4%), Caucasians (6–21 years, N = 30.3%), Hispanic (6–21 years, N = 21.2 %), Asian (5–13 years, N = 1.01%), Other (18 years, N = 6.6%) | Cohort study | African American children had higher levels of global DNA methylation than children of other races/ethnicities | Multifactorial, but environmental factors can create heritable changes in gene expression | Discussed |
| **Chitrala et al. (2020), USA** | Identifying epigenetic alterations associated with metabolic syndrome in African Americans and White Americans | DNAm analysis among AAs (N = 225) and White (N = 233) adults in the US | Longitudinal Cohort study | DNAm distinction between African American and Whites | Multifactorial: genetic causes, specific to each race, but several genes influenced by poverty status | Not Discussed |
| **Conway et al. (2015), USA** | Evaluating DNA promoter methylation profiles in invasive breast tumours from the CBCS | African Americans (N = 216) and non-Africans or Whites (N = 301) | Population-based, case–control study | Epigenetic variation could contribute to differences in breast tumour development and outcomes between AAs and non- AAs. | Even if genetics is key not ruled out that epigenetic racial variation may be associated with lifestyle or environmental exposures | Not Discussed |
| **Daca-Roszak et al (2020), Poland** | Comparing DNA methylation differences between populations of European and Chinese ancestry using B-lymphocyte cell lines | Europeans and Chinese origin | Cross sectional study | DNA methylation may be used to distinguish European and Chinese populations | Knowledge still limited, but discrimination between populations (…) based on DNA methylation markers, is feasible | Not Discussed |
| **de Mendoza et al. (2018), USA** | Exploring how perceived racial discrimination influences DNA methylation (DNAm) among African Americans | 147 African American mothers in the US | Longitudinal Cohort | Perceived racial discrimination influences DNA methylation (DNAm) among African Americans (AAs). | Not Discussed | Not Discussed |
| **Demerath et al. (2015), USA** | Examining epigenetic modifications associated with elevated adiposity (measured by both BMI and WC), including DNA methylation variation | 2097 African American adults, N = 2377) Whites, 991 Whites, 648 women | Prospective cohort study | Neutral | DNA methylation and other features of the epigenome are also modifiable throughout the life course by environmental and behavioural exposures | Discussed |
| **Devaney et al. (2015), USA** | Examining DNA methylation alterations in prostate cancer disparity | African Americans (N = 10), Caucasians (N = 11) | Observational study | AA & CA, taken for granted undiscussed | Methylation changed with diet | Discussed |
| **Do et al. (2021), UK** | Assessing whether diet quality was associated with differential DNA methylation | 4355 women from the WHI, 571 identical twins from the TwinsUK cohort | Longitudinal Cohort | Differently methylated sites in AA | Diet quality was significantly associated with differential DNA methylation | Not Discussed |
| **Enokida et al. (2005), UK** | Studying role of methylation in pathogenesis of PC, | 291 PC (Asians = 170; African Americans = 44; Caucasian = 77) and 172 benign prostate hypertrophy samples (BPH) (Asian = 96 African-American = 38) Caucasian = 38) | Longitudinal Cohort | Hypermethylation progresses according to different ethnic origin.in AA Caucasian & Asians, | The incidence of PC may be influenced by epigenetic events such as environmental factors including diet.  The incidence of PC may be influenced by epigenetic events such as environmental factors including diet. | Discussed |
| **Fraser et al. (2012), USA** | Investigating whether the genetic control of polymorphic DNA methylation is population-specific | Africans and one European population data from the (HapMap Project). [Canada, China (including Hong Kong), Japan, Nigeria, the United Kingdom, and the United States] | Longitudinal cohort studies | DNA methylation is highly divergent between populations (African and European) | a combination of differences in allele frequencies and complex epistasis or gene × environment interactions. | Not Discussed |
| **Galanter et al. (2017), USA** | Exploring differences in methylation between Latino sub-groups | 573 participants’ from diverse Latino ethnic sub-groups in the US (Africans, Native Americans and Europeans) | Longitudinal cohort studies | Neutral, assume existing classification | Differences in methylation (…) reflecting social and environmental influences -- not captured by ancestry | Not Discussed |
| **Giri et al. (2017), India** | Performing a comparative analysis of methylome from five different ethnicities. | [Caucasians (N = 10), African Americans (N = 10), Japanese (N = 10), Indo-Europeans (N = 10) and Dravidians (N = 10)] | Longitudinal cohort studies | Yes, methylation differences in different ethnicities | Methylation patterns correlate with dietary, cultural and demographical divergences across different ethnic groups. | Not Discussed |
| **Giuliani et al. (2016), USA** | Investigating the dynamics that create epigenetic diversity between and within different human groups | Americans of European ancestry (N = 96) ,Americans of African ancestry (N = 96) and Han Chinese Americans (N = 96) | Longitudinal cohort studies | European and African populations: extensive epigenetic differences between populations could have genetic or environmental underpinnings - or a combination of both | Yes, plasticity of DNAm | Not Applicable |
| **Heyn et al. (2013), USA** | Examining differential methylation contribution to variability in disease | Caucasian-American (N = 96), African American (N = 96), and Asian Americans (N = 96) | Populations base case-control | Yes, three human populations (Caucasian-American, African American, and Han Chinese- American) differentially methylated | Specific epigenetic (non genetic) contribution to natural human variation; that environmental variations between populations (e.g., diet preferences) could have influenced the epitype of germline cells, | Not Discussed |
| **Horvath et al. (2016), USA** | Comparing “epigenetic aging” by gender and race/ethnicity | African ancestry Caucasian; Hispanic; Han Chinese and Tsimane | Longitudinal cohort | Yes, different intrinsic epigenetic age correlates to different race/ethnicity | biological aging that incorporates genetics and the environment and | Not Discussed |
| **Jhun et al. (2021). USA** | Examining the association between DNA methylation and disease in a multi-ethnic sample | Europeans (N = 11,114), African Americans (N = 4,452), and Hispanics (N = 699 ) | Cohort study | Yes, Europeans, African Americans, and Hispanics | Complex traits are a manifestation of not only genetic but also environmental factors which in part express themselves through the epigenetic modification of DNA in all cell types | Not Discussed |
| **Kader et al. (2020), South Africa** | Testing for the reliability of methylation between racial groups for use in forensic testing. | 110, 4 major racial groups in South Africa (Blacks, Whites, Coloureds and Indians) | Cross sectional study | Neutral | Attributes some variation to genetics. However, external factors such as age, diets, lifestyle, ethnicity, can influence methylation patterns. | Not Discussed |
| **Lapato et al. (2018), USA** | Exploring both socio-environmental determinants of health and DNA methylation to understand racial disparities in pre-term birth | 177 women, African (N = 89) or European Americans (N = 88 ) in the US | Longitudinal cohort | Neutral | Multifactorial, but emphasis on causal relationships between environmental exposures and both DNAm and perinatal outcomes. | Not Discussed |
| **Lara et al. (2020), USA** | Exploring racial disparities in cancer outcomes | African American (AA) and European American (EA) patients | Longitudinal cohort | Yes, findings demonstrate that “African Americans have a higher risk of death for many cancer types compared with European Americans” | Multifactorial, genetic and epigenetic contributions highlighted | Not Discussed |
| **Li et al. (2020), China** | Performing a pan-cancer clinical and epigenetic molecular analysis of outcomes in African American (AA) and European American (EA) patients | Chinese men (N = 200) , 13 Western cohorts and Chinese cohort (N = 2,554) | Longitudinal cohort | Chinese patients were markedly distinct from those of Western cohorts: | Causation mechanism not discussed | Not Discussed |
| **Lynn et al. (2019), USA** | Identifying differences in DNA methylation levels in breast milk by race and lactation duration as risk factors for breast cancer | Black (N = 57) and White (N = 82) lactating women | Cross sectional | Neutral | Race and lactation duration may affect DNA methylation with effects on morbidity | Discussed |
| **McKennan et al. (2021), USA** | Exploring the relative contribution of genetic and environmental factors to race associated to DNA methylation patterns | 196 Children of primarily self-reported Black (N = 147) and Hispanic ethnicities (N = 39), white (N = 1), Mixed (N = 1) Other(N = 2) | longitudinal study | Neutral, assume existing classification | DNA methylation patterns are primarily driven by genetic factors and not as sensitive to environmental exposures | Not Discussed |
| **Mozhui et al. (2015), USA** | Examining ancestry-specific methylation patterns implicated in population differences in risk factors | African American (N = 112) and European American (N = 91) | Cohort study | Population differences [African American (AA; N = 112) and European American (EA; N = 91)] in DNA methylation patterns are remarkably stable | Driven by nutrition; divergence between AA and EA in methylation may be due to a combination of environmental and genetic factors | Not Discussed |
| **Needham et al. (2015), USA** | Examining associations between SES and gene specific DNAm in a large, population based sample of US adults | 1,264 nonHispanic white, African American, and Hispanic participants | longitudinal study | Non-Hispanic white, African American, and Hispanic | More work is needed to disentangle the social, environmental, psychological, and behavioral mechanisms underlying associations between SES and DNA methylation | Not Discussed |
| **Okosun et al. (2000), USA** | Determining relation between adiposity and BW | White (N = 759), Black (N = 916) and Hispanic (N =813) American children | longitudinal study | Yes, White, Black and Hispanic American children aged 5±11 y. | May be due to fetal programming, parsing of different factors yet to be established | Discussed |
| **Paredes-Céspedes et al. (2021), Mexico** | Investigating methylation profiles in indigenous Huichol communities | Ingenuous population (N =140), Mestizos populations (N = 47) | Cross sectional | Difference was found in the indigenous population when compared to a Mestizo population previously studied by our group | This difference might be due to the influence of the genetic admixture and differing dietary and lifestyle habits, including pesticide exposure, diet. | Not Discussed |
| **Park et al. (2018), USA** | Exploring racial differences in effects of cigarette smoking | 612 smokers from: whites (n = 204), Native Hawaiians (n = 205), and Japanese Americans (n = 203). | Observational | Epigenome of Native Hawaiians appeared to be differentially sensitive to the effects of internal smoking dose | Yes: Smoking directly affecting epigenome but differently by ethnicity | Not Discussed |
| **Pepin et al. (2021), USA** | Studying epigenetic differences among patients with end-stage heart failure (HF) | Multicohort African American (AA)  Caucasian American (CA) patients (n=42) | Cohort-based study | Critical, but still relying on AA/CA classification Yes: Black-white dichotomy | Multifactorial: Racism included as potential cause of epigenetic differences  Altered DNA methylation  may contribute to ethnic differences in body fat distribution and cardiometabolic risk. | Discussed |
| **Pheiffher et al. (2020), South Africa** | Investigating DNA methylation contribution to ethnic differences in body (adipose tissues) | Normal weight and obese black and white South African women (N=54) | Observational | Yes: Black-white dichotomy | Altered DNA methylation  may contribute to ethnic differences in body fat distribution and cardiometabolic risk. | Discussed |
| **Philibert et al. (2020), USA** | Testing whether array-based epigenetic aging indices were in fact tagging ethnicity and confounding comparisons of race/ethnicity | 203 newborns, African American (N = 112) and 91 White (N= 91)) | Cohort study | Neutral | No; Influence of cryptic ethnic-specific genetic influences | Not Discussed |
| **Rai et al. (2019), USA** | Identifying epigenetic differences and cancer risk factors | African American (N = 3), Caucasian counterparts (N = 3). | Cross sectional | Yes taken for granted, African‐American men (AA)  Caucasian men (CA). | Epigenetic changes are an inherent genomic property that appears early during development38 or acquired during life due to exposure to certain environmental factors | Discussed |
| **Rawlik et al. (2017), UK** | Explaining ethnic differences in colorectal cancer incidence | 132 admixed individuals from Colombia | Case-control study | Neutral | Potential role of direct environmental effects but still to be validated by further research | Not Discussed |
| **Rizzo et al. (2020), USA** | Determining if maternal obesity or diabetes mellitus during pregnancy result in a change in infant methylation | 69 American Hispanic population, (Hispanic Non-Hispanic, white, African American, Other) | Perspective study | Yes, differential DNA methylation in the fetal epigenome in a highly Hispanic population | How the fetal epigenome could be altered by the maternal environment; prenatal programming | Not Discussed |
| **Salihu et al. (2016), USA** | Identifying DNA methylation changes in relation to preterm birth, | “Black and non-black” mothers | Cross-sectional study | Yes, differentially methylated genes between black and nonblack  Newborns: lower levels of methylation among infants of black mothers. | Speculatively:  factors, such as diet, stress, and labor, mentioned | Discussed |
| **Song et al. (2015), USA** | Assessing differences by race for genome-wide DNA methylation and gene expression in healthy women with no prior history of breast cancer | European Americans (N = 61), African Americans (N = 22) | Longitudinal cohort | Differences in methylation and gene expression between EAs and Aas | Not clearly discussed | Not Discussed |
| **Straughen et al. (2015), USA** | Examining whether methylation may partially explain racial differences in BW | Non-Black (N = 66), Black (N = 21) | Cross-sectional study | Black infants had significantly higher IGF1 methylation than non-Black infants | Yes, epigenetic changes may mediate the effects of the prenatal environments | Not Discussed |
| **Tajuddin et al. (2019), USA** | Examining the association between DNAm and chronological age in relation to race, poverty, and sex, | Middle-aged AA (N = 244) and white (N = 243), men (N = 248), and women (N = 239) in the US | Prospective longitudinal study | Yes: “African Americans have more wide-spread methylation changes than whites”. | Multifactorial,  but cumulative effect of lifetime stress | Discussed |
| **Tehranifar et al. (2018), USA** | Investigating whether maternal smoking in pregnancy, independent of own personal active smoking, was associated with midlife DNA methylation in CpGs | Pregnant mothers of 89 adult women | Longitudinal cohort | Differences in methylation between African American women White and Hispanic women | Role for cigarette smoke exposure in altering DNA methylation | Discussed |
| **Tekola-Ayele et al. (2020), USA** | Identifying DNA methylation changes in placenta with impact on fetal development and adult life diseases | An ethnically diverse of cohort pregnant women (301)Hispanics (N = 102), Whites (N = 77 ), Blacks (N = 72), and Asians (N = 50) | Longitudinal cohort | Yes, based on self-identified race: 102 Hispanics, 77 Whites, 72 Blacks, and 50 Asians. | Multifactorial: Methylation changes “potentially due to cumulative environmental exposures (…) and genetic regulation” | Not Discussed |
| **Wang C et al. (2018), China** | Investigating susceptibility for diabetes in different ethnic groups | 24 Han (N = 12 ), Kazak (N = 6 ) groups and other (N = 6 ). | Observational studies | Yes, different methylation patterns in  two ethnicities. | No: Focus on genetic differences between the two populations | Not Discussed |
| **Wang X. et al. (2016), USA** | Elucidating epigenetic differences in racial disparities in colorectal cancer | African Americans (N = 6) and Caucasian Americans (N = 7) | Cross-sectional study | Yes, genes are differentially methylated in AA CRC compared to CA CRC | Multifactorial, socio-economic factors and epigenetic differences as drivers of racial disparity | Discussed |
| **Wiley et al. (2013), USA** | Investigating ethnic differences in epigenetic changes among patients with systemic lupus erythematosus | 224 Participants. African American and European American lupus and non-lupus women | Cross-sectional study | Differential DNMT associated with epigenetic changes between AA and EA | Admitted, but speculative: early life environmental influences, such as exposure to infectious agents, can lead to modula-tion of DNMTs expression | Discussed |
| **Workalemahu et al. (2021), USA** | Exploring whether maternal cardiometabolic status and ethnic differences in placental epigenetic age acceleration | 301 pregnant women from 4 race/ethnic Native American ancestry, African ancestry, Hispanics, and l East Asian ancestry  groups | longitudinal study | Neutral, based on self-reported race/ethnicity but utility of genetic ancestry as a modifying factor in epigenetic association studies | Multifactorial, maternal obesity to exert adverse in-utero influence on placental pathology | Not Discussed |
| **Zaghlool et al. (2018), Qatar** | Elucidating the molecular pathways that connect methylation to disease or lifestyle factors | 359, Arab and Asian ethnicity | Population-based cohort study | a diabetes cohort including Arab and South Asian ethnicities (Qatar) | As new insight: multiomics- associated CpG methylation is a consequence of the underlying disease pathway or an environmental insult. | Not Discussed |
| **Zhu et al. (2016), USA** | Understanding the influence of vitamin D deficiency on epigenome | 454 Caucasians (N = 263) and African Americans (N = 191) | Observational cohort | African Americans are generally at a hypomethylated state compared to Caucasians | Yes: Impact of nutrition on epigenome | Discussed |

**Supplemental Table 1. Characteristics of Included Studies**
