# Supplementary material for: A biosocial return to race? A cautionary view for the postgenomic era
Source: Am J Hum Biol. 2022 Mar 11;34(7):e23742. doi: 10.1002/ajhb.23742 (PMC9286859; doi:10.1002/ajhb.23742)
Supplement: Supplementary file 2 — Table S2 Supplementary Information. [file AJHB-34-0-s002.docx]

| **Participants and Study Characteristics** | | |
| --- | --- | --- |
| **Participants and study level** | **Number** | **Percentage** |
| **Sex** | | |
| Adult Women only | 13 | 26.5% |
| Adult Men only | 5 | 10.2% |
| Mix sex (Adults only) | 26 | 53.1% |
| Infants and children | 5 | 10.2% |
| **Countries** | | |
| USA | 38 | 77.6% |
| UK | 3 | 6.1% |
| China | 2 | 4.1% |
| South Africa | 2 | 4.1% |
| Poland | 1 | 2.0% |
| Mexico | 1 | 2.0% |
| Qatar | 1 | 2.0% |
| India | 1 | 2.0% |
| **Study designs** | | |
| Longitudinal | 33 | 67.3% |
| Cross sectional | 13 | 26.5% |
| Case Control | 3 | 6.1% |
